# Optimization, Characterisation and Pharmacokinetic Studies of Mucoadhesive Oral Multiple Unit Systems of Ornidazole

**DOI:** 10.3797/scipharm.1003-03

**Published:** 2010-12-02

**Authors:** Govind S. Asane, Yamsani Madhusudan Rao, Jaykrishna H. Bhatt, Karimunnisa S. Shaikh

**Affiliations:** 1 Department of Pharmaceutics, Pravara Rural College of Pharmacy, Loni- 413736, India; 2 University College of Pharmaceutical Sciences, Kakatiya University, Warangal- 506 009, India; 3 Department of Pharmaceutics, Poona College of Pharmacy, Pune-411038, India

**Keywords:** Bioavailability studies, Gastrointestinal transit, Factorial design, Mucoadhesion

## Abstract

The objective of the present study was to investigate the applicability of matrix type mucoadhesive oral multiple unit systems (MUS) for sustaining the release of ornidazole in the gastrointestinal tract (GIT). The MUS were prepared by ionotropic gelation method using chitosan and hydroxypropyl methyl cellulose K4M (HPMC K4M) according to 3^2^ factorial designs and were evaluated *in vitro* and *in vivo*. The particle size length ranged from 0.78 to 1.30 mm and breadth from 0.76 to 1.30 mm, respectively. The entrapment efficiency was in range of 80 to 96%. The rapid wash-off test was observed faster at intestinal pH 6.8 as compared to acidic pH 1.2. The fluoroscopic study revealed the retention of MUS in GIT for more than 5 hours. The pharmacokinetic parameters C_max_, T_max_, mean residence time (MRT) and area under curve (AUC) of developed MUS were found to be improved significantly (p<0.05) when compared with marketed immediate release tablets each containing 500 mg of drug. This study demonstrates that the MUS could be a good alternative to immediate release tablets to deliver ornidazole and expected to be less irritant to gastric and intestinal mucosa.

## Introduction

Oral administration of drugs by means of controlled release delivery systems should ideally enable to obtain the required plasma levels and to keep them steady for a prolonged period of time. Unfortunately, this ideal therapeutic target cannot systemically be achieved [1]; in spite of the progresses accomplished today in formulation and control of drug release kinetics from such type of dosage forms. The main limitations come from the variability of gastrointestinal transit time and from the non-uniformity of drug absorption throughout the gastrointestinal tract (GIT) [2]. These physiological limitations could be overcome by prolonging the gastric residence time of the dosage forms. Therefore, it would be beneficial to develop a sustained release formulation which remains at the absorption site for an extended period of time. Several approaches have been immersed to prolong the gastric residence time of the dosage forms at the absorption site and one of these is the development of oral controlled release bioadhesive system [3, 4]. The purpose of designing MUS dosage form is to develop a reliable formulation that has all the advantages of a single unit formulations and yet devoid of the danger of alteration in drug release profile and formulation behaviour due to unit to unit variation, change in gastro-luminal pH and enzyme population. A generally accepted view is that MUS perform better *in vivo* than single unit systems, as they spread out throughout the length of the intestine causing less irritation, enjoy a slower transit through the GIT and give a more reproducible drug release [5]. MUS, in general, have the potential to be used for targeted and controlled release drug delivery; but coupling of bioadhesive properties to MUS has additional advantages, e.g. efficient absorption and enhanced bioavailability of the drugs due to a high surface to volume ratio, a much more intimate contact with the mucus layer and specific targeting of drugs to the absorption site [6].

Ornidazole, a water-soluble drug is widely used as an anti-amoebic and antiprotozoal as well as to prevent recurrence of peptic ulcer disease caused by *H. Pylori.* Ornidazole has better oral bioavailability more than 90%, gets absorbed from small intestine with MIC of 0.25 mg/l [7]. The adverse effects of ornidazole are urticaria, pruritus, flushing, dry mouth, feeling of pelvic pressure, vertigo, headache, ataxia and insomnia which involve the GIT and nervous system, especially with high doses. Reduction of side effects while prolonging its action by using controlled release of oral dosage forms is highly desirable for such category of drugs [8, 9].

Chitosan, the n-acetylated product of the polysaccharide chitin, is gaining increasing importance in drug delivery to the GIT and also enhances the penetration of macromolecules across the intestinal and nasal barriers [10]. Sodium alginate, an anionic polysaccharide forms a cured gel matrix in the presence of calcium. Calcium alginate is able to incorporate drug within its gel matrix, and thus acts as a vehicle for the sustained release of orally administered drugs [11]. Chitosan and sodium alginate were selected owing to their good biocompatibility, non-toxicity and biodegradability. Statistical optimization designs have been previously documented for the formulation of many pharmaceutical oral solid dosage forms. Additionally, it is a powerful, efficient and systematic tool that shortens the time required for the development of pharmaceutical dosage forms [12].

Side effects, mainly at the gastric level, are well known, following oral administration of an ornidazole drug. Therefore, the efforts of many researchers have been concerned to solve these problems through a variety of techniques of protection of the gastric mucosa or alternatively to prevent the ornidazole release in this district. The objective of the present study was to evaluate the potential utility of natural biopolymers such as chitosan in combination with HPMC in sustaining the release of ornidazole in GIT. We also investigated the possible applicability of chitosan treated matrix type systems as sustained release system. We prepared MUS of dispersed chitosan and HPMC using aqueous solvents and evaluated *in vitro* and *in vivo*.

## Results and Discussion

MUS of ornidazole consisting of chitosan and HPMC K4M in varying compositions could be prepared by the orifice-ionic gelation process by 3^2^ factorial designs. The data analysis of parameters obtained from various batches for particle size distribution, drug entrapment efficiency and drug release were subjected to multiple regression analysis. Positive sign of the term indicates positive (additive) effect, while negative sign indicates negative (antagonistic) effect of the factor on the response. This design was selected as it provides sufficient degrees of freedom to resolve the main effects as well as the factor interactions. The data analysis was done by using “PCP Disso V3” software (IIPC, PCP, Pune, India) and fitted in equation:

Eq. 1. $$\text{Y}=\beta_{0}+\beta_{1}{\text{X}}_{1}+\beta_{2}{\text{X}}_{2}+\beta_{11}{\text{X}}_{1}^{2}+\beta_{22}{\text{X}}_{2}^{2}+\beta_{12}{\text{X}}_{1}{\text{X}}_{2}$$

Where, Y represents measured response; X levels of factors, and β, coefficient computed from the responses of the formulations.

### Determination of particle size

The results of evaluation of the micromeritic properties are shown in Table 1. The length and the breadth of MUS ranged from 0.78 ± 0.25 to 1.30 ± 0.21 mm and 0.76 ± 0.45 to 1.30 ± 0.21 mm, respectively. The MUS of ORZ-4, ORZ-5 and ORZ-6 were found to be discrete, spherical, free-flowing and of the mono-lithic matrix type. The batches of ORZ-7, ORZ-8 and ORZ-9 were found to be slightly irregular while others, ORZ-1, ORZ-2 and ORZ-3 were found to be very irregular in shape. The regression equation of particle size for MUS was:

Eq. 2. $${\text{Y}}_{1}=0.9983+0.1432\;{\text{X}}_{1}+0.1478{\text{X}}_{2}\;\;\;\;\;\;\;\;\;\;\;\;\;\;\;\;\;\;\;({\text{r}}^{2}=0.886)$$

**Fig. 1.**
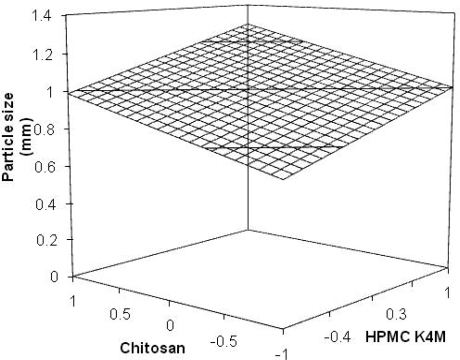
Response surface graph for the effect of variables on particle size.

The multiple regression data obtained for particle size indicated positive influence of the variables on the study, HPMC K4M exhibiting predominant effect than chitosan. The increasing viscosity of the solution caused by increasing both polymers concentration increases the droplet size and consequently the particle size. It also may be due to the fact that increased concentration of polymers, increases the crosslinking, and hence the matrix density of the MUS is also increased, which further results in the increase of the particle size of the MUS [13, 14]. The influence of the variables on the particle size of MUS was further elucidated using the response surface graph as shown in Fig. 1. The linear model generated for the geometric particle size was found to be significant with an *F*-value of 23.33 (*p*<0.05).

### Drug entrapment efficiency

The drug entrapment efficiency was in the range of 80 ± 3.45% to 96 ± 3.27% (Table 1). The regression equation of encapsulation efficiency for MUS was:

Eq. 3. $${\text{Y}}_{2}=94.3333-8.1667{\text{X}}_{1}{\text{X}}_{1}\;\;\;\;\;\;\;\;\;\;\;\;\;\;\;\;\;\;\;({\text{r}}^{2}=0.577)$$

**Fig. 2.**
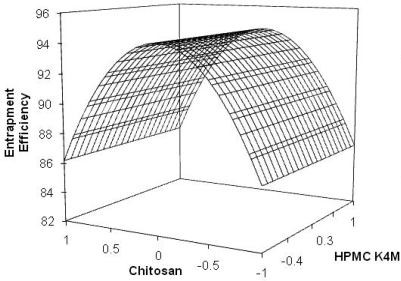
Response surface graph for the effect of variables on entrapment efficiency.

The negative result can be attributed to the concentration of chitosan on encapsulation efficiency. The decrease in entrapment efficiency with increase in chitosan concentration could be due to the increased binding of the main groups of the drug to the added chitosan [15]. The influence of the variables on the entrapment efficiency of the MUS was further elucidated using the response surface graph (Fig. 2). The linear model generated for the entrapment efficiency was found to be significant with an *F*-value of 9.57 (*p*<0.05). All the formulations showed high entrapment efficiency irrespective of the polymer concentration.

### Swelling studies

**Fig. 3.**
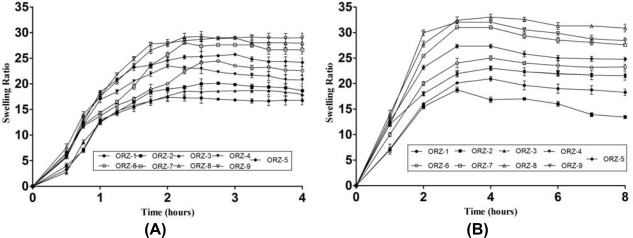
Swelling ratio of ornidazole MUS in (A) 0.1 N HCl pH 1.2 (B) Phosphate buffer pH 6.8

Swelling of MUS was influenced by the environmental pH. The swelling ratio of MUS in 0.1N HCl ranged between 16.70 ± 0.29 to 29.01 ± 0.19 (Fig. 3A). The MUS of ORZ-5 and ORZ-6 batches displayed the highest swelling in 0.1N HCl, which might be due to high concentration of HPMC K4M which swells rapidly in acidic medium. The swelling in phosphate buffer (Fig. 3B) ranged between 16.82 ± 4.57 to 33 ± 8.86. Increase in HPMC K4M and chitosan concentration resulted in slightly decreased swelling behaviour in phosphate buffer. This may be due to insolubility of chitosan in alkaline conditions. The properties of the polymers were generally affected by their swelling behaviour, water uptake and hydration state [16]. Therefore, a slow swelling is a requisite to avoid the formation of an over hydrated form that loses its integrity before the drug release at the target. The intact nature of the MUS is required to maintain a slow drug release during their transit through GIT. The batches ORZ-6, ORZ-7 and ORZ-9 were found to remain intact, whereas ORZ-3, ORZ-5 changed their shape and remaining batches were totally disintegrated.

### Mucoadhesion testing by in vitro wash-off method

**Fig. 4.**
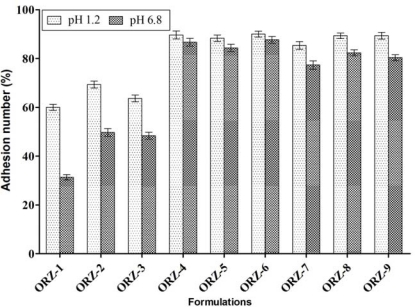
Mucoadhesion testing of different ornidazole formulations in pH 1.2 and pH 6.8 at 12 h (n=3).

MUS consisting of alginate, HPMC K4M and chitosan in different concentrations exhibited good mucoadhesive properties in the *in vitro* wash-off test as shown in Fig. 4. The wash-off was faster at intestinal pH (33 ± 1.00 to 90.33 ± 1.52) than at gastric pH (60 ± 2.00 to 90 ± 1.00). The rapid wash-off was observed at intestinal pH 6.8, a pH at which ionization of carboxyl and other functional groups of the polymer occurs leading to increase in their solubility and reduction of adhesive strength [17]. The adhesion number was found to be increased with increase in amount of both the polymers because more amount of polymer results in higher amount of free –NH_2_ groups, which are responsible for binding with sialic acid groups in mucus membrane and thus results in increase in mucoadhesive properties [18]. The initial inter-action between the polymer and the biological surface is through electrostatic interaction followed by mechanical interlocking of the polymer chains. Therefore, the surface charge density of polymers is important for electro-static behavior during the adhesion process [16]. The results of the wash-off test indicated that the MUS demonstrated fairly good mucoadhesive properties under acidic conditions.

### Drug release study

**Fig. 5.**
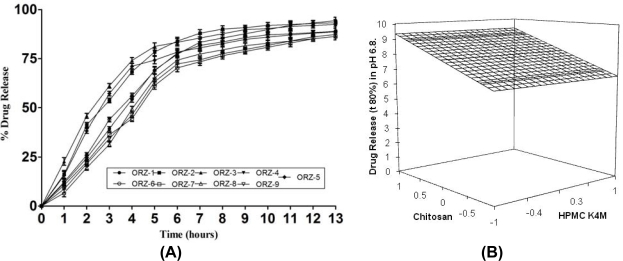
(A) In-vitro release profile of ORZ MUS in phosphate buffer pH 6.8. (B) Response surface graph for the effect of variables on t_80%_ of drug release.

The drug release analysis was performed by taking data of phosphate buffer pH 6.8 and complete dissolution of drug from the MUS occurred within 13h as shown in Fig. 5A. The drug release was initially rapid followed by steady state. The ideal drug release up to 12 h was shown by ORZ-6, ORZ-7, and ORZ-8. The regression equation for time taken to release the drug in phosphate medium was as follows:

Eq. 4. $${\text{Y}}_{3}=8.0070-1.3273\;{\text{X}}_{1}\;\;\;\;\;\;\;\;\;\;\;\;\;\;\;({\text{r}}^{2}=0.886)$$

The multiple regression equation revealed retarding effect of chitosan on drug release, which might have caused by slower penetration of medium in highly cross-linked MUS due to formation of a three dimensional gel structure leading to immobilization of water molecules inside it and slowing drug release in buffer [19]. When the amount of polymer is increased the crosslink density increases which causes barrier for drug diffusion and hence the rate of release decreases and t_80%_ increases. The influence of the variables on the t_80%_ of drug release was further elucidated using the response surface graph (Fig. 5B). The linear model generated for the geometric percent drug release was found to be significant with an *F*-value of 6.61 (*p* < 0.05). The figure reveals that drug release rate was slowed after 4 h. The batch ORZ-6 exhibited a high t_80%_ of 530 min and seems to be a promising candidate for achieving drug release up to 10 h. The model fitting of the *in vitro* release profile (Table 2) in different release kinetics models and comparison of the coefficient of determination (r^2^) showed that release data of ORZ-1, ORZ-2 and ORZ-6 to ORZ-9 obeys first order kinetic; whereas of ORZ-3 to ORZ-5 follows matrix model kinetics. To justify the results, power law was applied and from the diffusion coefficient value (n), it was found that ORZ-1, ORZ-2 and ORZ-4 to ORZ-9 formulations follow anomalous diffusion transport mechanism while ORZ-3 follows Fickian diffusion. This can be attributed to the fact that the drug release from the MUS did not follow uniform geometry; instead the drug got released through fractal rearrangements of polymeric chain. An ideal drug release up to 12 h was shown by ORZ-4, ORZ-5 and ORZ-6. The percent of the drug released for ORZ-6 at the end of 5,10 and 12 hours was 68,87 and 93%, respectively. The drug release was highly extended from ORZ-7, ORZ-8 and ORZ-9 which contain higher concentration of HPMC K4M that forms a swollen gel with greater structural integrity and greater entanglement holding excess of medium.

### Gastrointestinal Transit (GI) behaviour

**Fig. 6.**
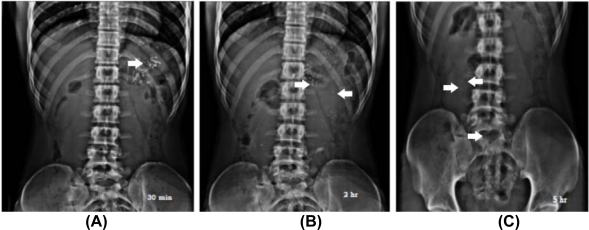
X-ray photographs recorded at (A) 0.5 h, (B) 2 h and (C) 5 h after oral administration of blank formulation of ORZ-6 in human volunteers.

As compared to the normal gastric residence time of the conventional formulations from 5 minutes to 2 hours, the residence time of ORZ-6 MUS was found to be improved. The fluoroscopic study revealed that the MUS remained in the stomach for 30–60 minutes and then passed into the upper intestinal tract where they stayed up to 3 h, followed by the small intestine up to 6 h and retained for longer time in the colon for more than 6 h. The administered formulation could not be detected after 24 h possibly due to the degradation of polymers in the colon and/or evacuation during the passage of the bowel. X-ray photographs obtained at 5 h showed maximum number of MUS in the small intestine when compared to transit time of small intestine of 3–4 h, as most of the drugs are absorbed through it due to its huge surface area [20] as shown in Fig. 6. The tested formulation could be useful for the sustained delivery of ornidazole as it stayed at different sites and spread over a larger area of the GIT. There was no significant effect of food in the transit of the MUS with mucoadhesive polymers.

### In vivo bioavailability study

**Fig. 7.**
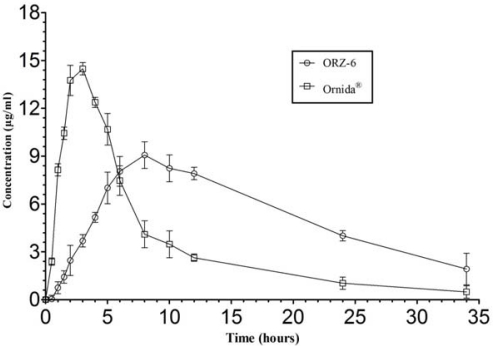
Mean serum levels of ornidazole after oral administration of formulation ORZ-6 and Ornida^®^ 500 mg tablets. Each point represents mean value ± standard deviation (n =8).

The ORZ-6 formulation was selected for bioavailability studies due to its good swelling property in acidic and alkaline conditions and also possessing good bioadhesive property in both the media, while Ornida^®^ IR 500 mg tablet was selected because no sustained release formulation of ornidazole was available in Indian market. The values of pharmacokinetic parameters were tested for equality of variance; on acceptance of the hypothesis, paired *t*-test was used to test the significance of the observed difference in pharmacokinetic parameters; else, the *t*-test with unequal variance was used to test the significance. The mean plasma concentrations of ornidazole at each time point following administration of ORZ-6 and Ornida^®^ are shown in Fig. 7 and the pharmacokinetic parameters are listed in Table 3.

The peak concentration (C_max_) of Ornida^®^ tablet and ORZ-6 MUS was 14.48 ± 1.27 and 9.03 ± 0.73μg/ml, respectively with a significant difference of (P<0.0001) with each other. The time to reach peak concentration (T_max_) of Ornida^®^ tablet and ORZ-6 MUS was 3.00 ± 0.16 and 8.00 ± 0.22 h, respectively with significant difference of (P<0.0001) with each other. The extent of absorption is a key factor of the formulation and therefore the absorption rate constant (k_a_) and AUC are important parameters for analysis in a comparative bioavailability study. Ornidazole was found absorbed at a faster rate resulting in a sudden spike of the drug level in plasma after oral administration of IR tablet. Though there was a delay in the appearance of the drug after the oral administration ORZ-6 MUS, similar spike in the drug levels was observed after 3 h. The absorption rate constant of ORZ-6 was 0.73±0.004 (1/h) when compared to Ornida^®^ with 5.32 ± 1.48 (1/h) with a significant difference of (P<0.0001) with each other. The mean elimination half-life for ornidazole following oral ingestion of IR tablet and ORZ-6 MUS were 5.36 ± 0.45 and 6.83 ± 1.15 h, respectively which were not significantly different. After administration of Ornida^®^ tablet and ORZ-6 MUS, AUC_0–24_ attained was 114.49 ± 12.88 and 170.68 ± 11.93 μg-h/ml, while, the AUC_total_ was 120.78 ± 24.32 and 200.71 ± 45.92μg.h/ml, respectively (P<0.0033). The MRT of Ornida^®^ tablet and ORZ-6 MUS was 10.46 ± 1.20 h and 20.13 ± 2.83 h, respectively (P<0.0063). The C_max_, T_max_ and AUC_0-t_ obtained with ORZ-6 MUS and Ornida^®^ tablet when compared with paired *t* -test showed significant difference (P<0.05) between the two formulations. This difference may be due to the reason that one product is administered as immediate release tablet and other as gastro retentive sustained release formulation. Thus, the bioavailability was improved as compared to immediate release marketed product. This also establishes the fact that the developed ORZ-6 formulation was effective and better than the marketed tablet dosage form. Higher bioavailability with same dose and showing sustained effect can be considered as better qualities of the developed formulations.

## Experimental

### Materials

Model drug ornidazole and metronidazole (internal standard) were gift samples from J. B. Chemicals Ltd, Ankleshwar, India. Chitosan (degree of deacetylation of 85%) was gifted by Central institute of fisheries technology, Kochi, India. Hydroxypropyl methylcellulose K4M (HPMC K4M, having a viscosity of 50 cps in a 2% w/v aqueous solution at 20°C) was kindly supplied by Colorcon Asia Pvt. Ltd. Sodium alginate (SK Fine Chem, Mumbai, India) and calcium chloride (Qualigens, Mumbai, India) were procured from commercial sources. Marketed ornidazole 500mg tablets (Ornida^®^ IR) used as reference product for bioavailability studies were purchased from local market (Loni, India). All other reagents and solvents used were of analytical grade procured from Merck, Mumbai, India.

## Methods

### Preparation of MUS by ionic gelation method [21]

Sodium alginate (2%w/v), HPMC K4M and chitosan in varying composition (Table 1) were dissolved in purified water 100ml to form a homogeneous polymer solution. The active substance ornidazole (2.0 g) was added to the polymer solution and mixed thoroughly with a stirrer to form a viscous dispersion. The resulting dispersion was then added manually dropwise into slowly agitated 100ml calcium chloride solution (2.5%w/v) through a syringe having a needle of size 16G. The added droplets were retained in the calcium chloride solution for 15 minutes to complete the curing reaction and to produce spherical rigid MUS. The MUS were collected by decantation and the product thus separated was washed repeatedly with 2x100ml volumes of de-ionized water and dried at room temperature for 12 h.

### Optimization by Factorial design experiments

A full 3^2^ factorial design was constructed and conducted to optimise the levels of the independent variables chitosan (X_1_) and HPMC K4M (X_2_). The dependent variables selected for the study were particle size (Y_1_), drug entrapment efficiency (Y_2_) and time taken for 80% of drug release (t_80%_) (Y_3_). The coded and actual value of variables for each batch and the experimental design are given in Table 1. The range of a factor was chosen in order to adequately measure its effect on the response variables.

### Determination of Particle Size

Twenty five MUS from each batch were subjected to determination of particle size by digital micrometer (Mitutoyo, Japan). Using the frequency data, the log normal distribution on a probability scale was plotted and the geometric mean diameter and the geometric standard deviation were calculated.

### Drug entrapment efficiency

Drug loaded MUS (50 mg) were placed in 250 ml conical flask containing 100 ml of 0.1N HCl solution in water. The MUS were stirred to release ornidazole completely. These solutions were centrifuged and filtered through a 0.45 μm membrane filter. The filtrate was then sufficiently diluted with 0.1N HCl solution. Aliquots of these solutions were subjected in triplicate to ultraviolet spectroscopy at 254 nm against 0.1N HCl solution as blank.

Eq. 5. $$\text{Microencapsulation}\;\text{efficiency}=\frac{\text{Estimated}\;\text{percentage}\;\text{drug}\;\text{content}}{\text{Theoretical}\;\text{percentage}\;\text{drug}\;\text{content}}\times 100$$

### Swelling studies

Known quantities of MUS (100 mg) of different batches were placed in 10 ml volumetric graduated measuring cylinders containing 0.1 N HCl pH 1.2 for 4 h and phosphate buffer pH 6.8 for 8 h. The length of MUS in graduated measuring cylinder was measured at appropriate time intervals. The studies were performed in triplicate and average volume of MUS was calculated. The relative swelling of MUS was calculated as percentage of the volume at time 0.

### Mucoadhesion testing by in vitro wash-off method

The mucoadhesive property of MUS was evaluated by an *in vitro* adhesion testing method known as the wash-off method as reported by Lehr et al [22]. The mucoadhesive property of the MUS was compared within the batches. The freshly excised pieces of intestinal mucosa of sheep was obtained from slaughter house and stored in Tyrode solution. The pieces (2×2 cm) were mounted onto glass slides (3×1 inch) with cyanoacrylate glue. Two glass slides were connected with a suitable support. About 25 (n=3) MUS were spread on to each wet rinsed tissue specimen and immediately thereafter the support was hung on to the arm of a USP tablet disintegrating test machine. When the disintegrating test machine was operated, the tissue specimen was given a slow, regular up and down movement in the test fluid at 37° ± 0.5°C contained in a 1 litre vessel of the machine. At the end of 30 min, at the end of 1 h and at hourly intervals up to 12 h the machine was stopped and the number of MUS still adhering to the tissue was counted. The test was performed at both gastric pH 1.2 and intestinal pH 6.8. It is assumed that as the adhesion strength increases, the adhesion number also increases. The adhesion number (N_a_) is defined as the ratio between the number of particles (N) remaining after the application of a certain detachment force and the number of particles (N_o_) originally present on the test surface. The adhesion number is often expressed as a percentage.

Eq. 6. $$N_{a}=\frac{N}{N_{0}}\times 100$$

### Drug release study

*In vitro* drug release from MUS was studied by means of a USP type II dissolution test apparatus (EDT-06 L, Electrolab, India) in phosphate buffer pH 6.8 for 13h at the speed of 100 rpm at 37±0.5^°^C. Samples of dissolution fluid were withdrawn through a cellulose nitrate filter (0.45μm) at different time intervals and were assayed spectrophotometrically at 319nm using a Shimadzu UV-150 double-beam spectrophotometer (Shimadzu Corporation, Japan). The drug release experiments were conducted in triplicate (n = 3). Analysis and model fitting [23] of data was done by using “PCP Disso V3” software (IIPC, PCP, Pune, India).

### Gastrointestinal Transit (GI) Behaviour

The GI transit behaviour of the formulation was visualized using fluoroscopy (low energy X-rays, Siemens Fluorovision, Germany) under the supervision of a radiologist. The study protocol was approved by the Institutional Ethical Committee (Kakatiya University, Warangal, India, and No-UCPSC/BA/2009-05). Three healthy human male volunteers of age and weight ranging from 23 to 29 years and 62 to 70 kg were selected for the study. A written consent was obtained from them. The MUS containing radio-opaque marker (barium sulphate) were prepared in a similar manner to formulation ORZ-6 by replacing the drug. The gelatin capsules containing 50 MUS each were administered to each subject with 200 ml of water after the subjects had fasted overnight. Lunch was provided 5 h after administration of radio-opaque formulation. During the experiments the subjects remained in a sitting or upright posture. All X-ray films were taken in anterior positions.

### In vivo bioavailability study

The bioavailability protocol was approved by the Institutional Ethical Committee (Kakatiya University, Warangal, India). Eight healthy male volunteers in the age group of 25–30 years (60–65 kg) participated in the study and all were non-alcoholics and non-smokers. The required biochemical tests were carried out to ensure that the volunteers were free from either liver or kidney dysfunction. None of the volunteers was on drug treatment 10 days prior to participation in the study. The nature and purpose of the study were fully explained to them. An informed written consent was obtained from every volunteer. They were allowed to withdraw from the study at any time, if they desire so. A crossover single dose study was followed. The volunteers were divided into two equal groups (group I and group II). Group I volunteers (n=8) received Ornida^®^ immediate release tablets (Batch no.175A019,Aristo Pharmaceuticals ltd, India) (dose 500 mg) whereas group II (n=8) volunteers received developed formulation ORZ-6 (dose 500 mg). The required amount of formulation ORZ-6 was dispensed into hard gelatin capsules. A light breakfast was provided after overnight fasting. After 30 minutes capsules and Ornida^®^ IR 500 mg tablets were administered to each subject with 200 ml of water. Lunch was provided 4 h after drug administration. Blood samples of 5 ml were collected at 0, 0.5, 1, 1.5, 2, 3, 5, 8, 10, 12, 24 and 34 h. The samples were allowed to clot and centrifuged at 3000 rpm for 10 minutes. Serum was separated and stored at −20°C until analysis.

### Estimation of Ornidazole in human serum

Analysis of ornidazole in human plasma samples was done by HPLC method reported by Y.S.R. Krishnaiah et al with slight modification [24]. The standard stock solution of ornidazole and metronidazole (internal standard) were prepared by dissolving 10 mg of each drug in 100 ml of double distilled water in separate volumetric flasks to get concentration of 100μg/ml. The stock solution of ornidazole was further diluted with double distilled water to get series of working standard solutions having concentration 0.6, 1.2, 3, 6, 12, 18, 24 and 30μg/ml. 12 ml of metronidazole stock solution was further diluted to 20 ml with double distilled water to get standard solution of concentration 60 μg/ml. 1 ml of each working standard solution of ornidazole (0.01–25 μg/ml) was transferred in a series of eppendorf tubes (Eppendorf-Netheler-Hinz, Hamburg, Germany) containing 1ml of human plasma separately. In each flask 0.5 ml solution of metronidazole (60 μg/ml) and 3.5 ml of acetonitrile was added for complete precipitation of proteins. The tubes were vortexed for 10 minutes on vortex mixer and then centrifuged for 10 min at 3500 rpm. The HPLC system was equipped with a pump (LC-10AT, Shimadzu, Japan), an injection port (Rheodyne, USA), a reversed phase C_18_ column (250 4.6 mm, 5 m, Phenomenex, USA) and a UV detector (SPD 10A, Shimadzu, Japan). HPLC mobile phase was composed of 10 mM KH_2_PO_4_ (pH 4.7): Acetonitrile (80: 20, v/v) at a flow rate of 1 ml/min with the detector wavelength set at 254 nm. The retention times of metronidazole and ornidazole were 5.48 and 9.15 minutes respectively. The peak area ratios of ornidazole to metronidazole were calculated and plotted against the respective concentrations of ornidazole to obtain the calibration curve. A good linear relationship was observed between the concentration of ornidazole and the peak area ratio of ornidazole to that of internal standard with a high correlation coefficient (r=0.999) in the range of 0.01–25 μg/ml. The method was found to be precise (intra- and inter- day variation was found to be less than 2%) and accurate (mean recovery 99.8%).

### Pharmacokinetics of MUS [25]

Peak plasma concentration (C_max_), the corresponding times at which these are reached (T_max_), the area under the serum concentration time curve (AUC) and Mean Residence Time (MRT) for individual subjects were calculated using KINETICA™ software (Inna Phase Corp., 2000). All the data was statistically analyzed using Sigmastat software package (Jandel Corp., California). Half-life (t_1/2_): The overall elimination rate constant (k_e_) was calculated from the slope of the terminal elimination phase of a semi-logarithmic plot of concentration versus time, after subjecting it to linear regression analysis. Assuming the elimination to be a first-order process: t_1/2_ =0.693/ke, where ke = −slope × 2.303, while absorption rate constant (k_a_) was obtained by assuming first-order kinetics using the equation: k_a_=4.61/t_a_, where t_a_ is the absorption time obtained from a semi-logarithmic plot of concentration versus time data [26]. Paired *t*-test was used for comparison of pharmacokinetic parameters and the difference was considered significant when p<0.05.

## Figures and Tables

**Tab. 1.** 3^2^ Factorial design: composition and characteristics of ORZ-loaded MUS.

**Formul.**	**Chitosan Amount (mg) (X_1_)/code**	**HPMC K4M Amount (mg) (X_2_)/code**	**Particle size (mm)**		**Entrapment Efficiency (%) Mean ± (SD)**
			**Length Mean ± (SD)**	**Breadth Mean ± (SD)**	
**ORZ-1**	1.00 (−1)	1.00 (−1)	0.78 ± 0.25	0.76 ± 0.45	88 ± 0.56
**ORZ-2**	1.00 (−1)	1.50 (0)	0.80 ± 0.14	0.80 ± 0.48	84 ± 0.50
**ORZ-3**	1.00 (−1)	2.00 (+1)	1.03 ± 0.23	1.01 ± 0.84	80 ± 0.45
**ORZ-4**	1.75 (0)	1.00 (−1)	0.87 ± 0.32	0.85 ± 0.23	92 ± 0.45
**ORZ-5**	1.75 (0)	1.50 (0)	0.89 ± 0.12	0.88 ± 0.36	96 ± 0.20
**ORZ-6**	1.75 (0)	2.00 (+1)	1.13 ± 0.22	1.13 ± 0.28	95 ± 0.20
**ORZ-7**	2.50 (+1)	1.00 (−1)	0.93 ± 0.21	0.94 ± 0.23	91 ± 0.20
**ORZ-8**	2.50 (+1)	1.50 (0)	1.22 ± 0.37	1.23 ± 0.23	90 ± 0.40
**ORZ-9**	2.50 (+1)	2.00 (+1)	1.30 ± 0.21	1.30 ± 0.21	84 ± 0.55

SD indicates standard deviation. Independent variables level: low (−1), medium (0), high (+1), HPMC-Hydroxypropyl methyl cellulose.

**Tab. 2.** Model fitting of *in-vitro* release data using correlation coefficient (r^2^) and n values.

**Formul.**	**Zero order**	**First order**	**Matrix**	**Hixson–Crowell**	**Peppas**	**n**

	**r^2^**					
ORZ-1	0.882	0.993	0.964	0.972	0.963	0.834
ORZ-2	0.712	0.964	0.955	0.915	0.933	0.615
ORZ-3	0.574	0.937	0.947	0.863	0.935	0.484
ORZ-4	0.763	0.949	0.968	0.915	0.967	0.562
ORZ-5	0.679	0.933	0.955	0.875	0.912	0.598
ORZ-6	0.831	0.965	0.964	0.943	0.954	0.743
ORZ-7	0.893	0.982	0.964	0.965	0.972	0.834
ORZ-8	0.888	0.964	0.957	0.944	0.958	0.953
ORZ-9	0.892	0.982	0.968	0.963	0.962	0.875

**Tab. 3.** Pharmacokinetics of ornidazole following oral administration of formulation ORZ-6 and Ornida^®^ 500 mg tablets (n=8).

**Pharmacokinetic parameters**	**Ornida^®^**	**ORZ-6**	**Significance**

	**Mean (±SD)**	**Mean (±SD)**	**Difference (p<0.05)**
C_max_ (μg/ml)	14.48 ± 1.27	9.03 ± 0.73	P<0.0001
T_max_ (h)	3.00 ± 0.16	8.00 ± 0.22	P<0.0001
AUC_0–24_ (μg.h/ml)	114.49 ± 12.88	170.68 ± 11.93	P<0.0033
AUC_Total_ (μg.h/ml)	120.78 ± 24.32	200.71 ± 45.92	P<0.0033
mean residence time (h)	10.46 ± 1.20	20.13 ± 2.83	P<0.0063
Terminal half-life, t_1/2_ (h)	5.36 ± 0.45	6.33 ± 1.15	P<0.05
Absorption time, t_a_ (h)	0.91 ± 0.05	5.83 ± 0.001	P<0.0001
Absorption rate constant, k_a_ (1/h)	5.32 ± 1.48	0.73 ± 0.004	P<0.0001

SD indicates standard deviation
